# Genetic susceptibility of intervertebral disc degeneration among young Finnish adults

**DOI:** 10.1186/1471-2350-12-153

**Published:** 2011-11-22

**Authors:** Anthi Kelempisioti, Pasi J Eskola, Annaleena Okuloff, Ulla Karjalainen, Jani Takatalo, Iita Daavittila, Jaakko Niinimäki, Roberto B Sequeiros, Osmo Tervonen, Svetlana Solovieva, Patrick YP Kao, You-Qiang Song, Kenneth MC Cheung, Danny Chan, Leena Ala-Kokko, Marjo-Riitta Järvelin, Jaro Karppinen, Minna Männikkö

**Affiliations:** 1 Oulu Center for Cell Matrix Research, Biocenter and Department of Medical Biochemistry and Molecular Biology, University of Oulu, Aapistie 7/P.O. Box 5000, 90014 Oulu, Finland; 2 Finnish Institute of Occupational Health, Finland; 3 Institute of Clinical Medicine, Department of Physical and Rehabilitation Medicine, University of Oulu, Oulu, Finland; 4 Department of Diagnostic Radiology, University of Oulu, Oulu, Finland; 5 Centre of Expertise for Health and Work Ability, Finnish Institute of Occupational Health, Helsinki, Finland; 6 Disability Prevention Center, Finnish Institute of Occupational Health, Helsinki, Finland; 7 Department of Biochemistry, The University of Hong Kong, Pokfulam, Hong Kong, China; 8 Department of Orthopaedics and Traumatology, The University of Hong Kong, Pokfulam, Hong Kong, China; 9 Connective Tissue Gene Tests, Allentown, Pennsylvania, USA; 10 Department of Public Health and General Practice, Institute of Health Sciences, University of Oulu, Oulu, Finland; 11 Biocenter Oulu, University of Oulu, Oulu, Finland; 12 National Institute of Health and Welfare, Oulu, Finland; 13 Department of Biostatistics and Epidemiology, School of Public Health, MRC-HPA Centre for Environment and Health, Imperial College, Faculty of Medicine, London, UK; 14 Finnish Institute of Occupational Health, Oulu, Finland

## Abstract

**Background:**

Disc degeneration (DD) is a common condition that progresses with aging. Although the events leading to DD are not well understood, a significant genetic influence has been found. This study was undertaken to assess the association between relevant candidate gene polymorphisms and moderate DD in a well-defined and characterized cohort of young adults. Focusing on young age can be valuable in determining genetic predisposition to DD.

**Methods:**

We investigated the associations of existing candidate genes for DD among 538 young adults with a mean age of 19 belonging to the 1986 Northern Finland Birth Cohort. Nineteen single nucleotide polymorphisms (SNP) in 16 genes were genotyped. We evaluated lumbar DD using the modified Pfirrmann classification and a 1.5-T magnetic resonance scanner for imaging.

**Results:**

Of the 538 individuals studied, 46% had no degeneration, while 54% had DD and 51% of these had moderate DD. The risk of DD was significantly higher in subjects with an allele G of *IL6 *SNPs rs1800795 (OR 1.45, 95% CI 1.07-1.96) and rs1800797 (OR 1.37, 95% CI 1.02-1.85) in the additive inheritance model. The role of *IL6 *was further supported by the haplotype analysis, which resulted in an association between the GGG haplotype (SNPs rs1800797, rs1800796 and rs1800795) and DD with an OR of 1.51 (95% CI 1.11-2.04). In addition, we observed an association between DD and two other polymorphisms, *SKT *rs16924573 (OR 0.27 95% CI 0.07-0.96) and *CILP *rs2073711 in women (OR 2.04, 95% CI 1.07-3.89).

**Conclusion:**

Our results indicate that *IL6*, *SKT *and *CILP *are involved in the etiology of DD among young adults.

## Background

Disc degeneration (DD) is a multifactorial condition that progresses with aging. With age, histological, metabolic and functional changes take place in the disc, making it stiffer and reducing its strength [1]. Although the events leading to DD are not well understood, the outcome of studies over the last decade has shown that genetic influence plays a large role in DD, together with environmental factors [2]. Known environmental risk factors for DD include body mass index (BMI) and heavy physical loading [3], although their effect is weak in comparison to heredity [4].

To date, several gene loci associated with human disc degeneration have been identified [5]. Variations in the genes involved in inflammation, extracellular matrix components, and protein metabolism have been reported as associating with DD [6,7]. The first polymorphisms associated with DD were two variations in the Vitamin D receptor gene (TaqI and FokI) [8]. Subsequently, associations with aggrecan (*ACAN*) and two collagen IX genes (*COL9A2*, *COL9A3*) were reported [9-11]. The structural components of the intervertebral disc have been an obvious choice as candidate genes, because variations in structural genes may render discs more vulnerable to mechanical loading, causing injuries to endplates and/or annulus fibrosus, leading to loss of disc function [5]. In addition, variants in genes encoding cytokines, such as interleukin-1 (*IL1*) and interleukin-6 (*IL6*), or degradative metalloproteinases (*MMP*s) have been reported as being associated with DD [12-15]. The genes of adipocytokines are also putative candidates for DD due to the association between DD and atherosclerosis [16]. However, of the reported associations, only a few have been verified in different ethnic populations [2].

Although genetic analyses have gained a more central role in the exploration of the underlying causes of DD, its etiology and pathogenesis still remain unclear. In general, genetic studies have suffered from small sample sizes and differences in the definition and characterization of DD. Here we investigated the association between relevant candidate gene polymorphisms and moderate DD in a well-defined and characterized cohort of young adults. Focusing on moderate DD phenotype manifesting at an early age will provide a better opportunity to determine the predisposing role of genetic polymorphisms.

## Methods

### Study population

The study population consisted of members of the 1986 Northern Finland Birth Cohort (NFBC 1986) with an expected date of birth between July 1, 1985 and June 30, 1986 (n = 9479) in the two northernmost provinces of Finland; Oulu and Lapland. The 2969 that lived within 100 km of the city of Oulu received a postal questionnaire in 2003. Altogether, 1987 responded, with a mean age of 18 (response rate 68%) and were invited to participate in a physical examination, which took place in 2005-2006 at a mean age of 19. The participants of the examination (n = 874) were invited to participate in lumbar spine magnetic resonance imaging (MRI) [17]. Our study population consisted of 538 subjects with both DNA sample and lumbar spine MRI. The Ethics Committee of the Northern Ostrobothnia Hospital District approved the study plan, and the study was performed according to the Declaration of Helsinki. Written informed consent was obtained from all individuals.

### Magnetic resonance imaging

MRI scans were performed using a 1.5-T unit (Signa, General Electric, Milwaukee, WI) and phased array spine coil (USA Instruments, Aurora OH, USA) with the imaging protocol of a sagittal T1-weighted (440/14 [repetition time msec/echo time msec]) spin echo, a sagittal T2-weighted (3960/116) fast spin echo (FSE), and the axial T2-weighted FSE (5160/107) of the entire lumbar spine.

The degree of DD was graded from T2-weighted images using a modified Pfirrmann classification: Grade 1 (normal shape, no horizontal bands, distinction of nucleus, and annulus is clear), Grade 2 (nonhomogeneous shape with horizontal bands, some blurring between nucleus and anulus), Grade 3 (nonhomogeneous shape with blurring between nucleus and anulus, anulus shape still recognizable), Grade 4 (nonhomogeneous shape with hypointensity, anulus shape not intact and distinction between nucleus and anulus impossible, disc height usually decreased), and Grade 5 (same as Grade 4 but collapsed disc space) [17,18]. In the modified Pfirrmann classification, hyperintensity or isointensity of the intervertebral disc to cerebrospinal fluid (CSF) ratios were not used as criteria for Grades 1 through 3, due to the fact that CSF was hyperintense compared to the discs in all the MR sequences. Moderate DD was defined as Grade 4 degeneration at one or more lumbar levels, or Grade 3 degeneration at three or more levels. Subjects with Grade 1 and Grade 2 findings and no other pathology in MRI were defined as the reference group, i.e. subjects without DD. Information on inter-rater reliability and comparison of the MRI study population and the rest of the survey respondents have been published elsewhere [17].

### Candidate gene and single-nucleotide polymorphism (SNP) selection

At the time of the study conception we listed all putative DD candidate genes and polymorphisms. The current genotyping assay of choice is designed for SNP genotyping thus excluding other types of genetic variations from this study. After testing different polymerase chain reaction (PCR) multiplexes we were able to include nineteen SNPs in five separate multiplexes. These SNPs were distributed in 16 different genes (Table 1). Due to the structure of the DNA sequences at the SNP sites we were not able to include all possible polymorphisms in the analyses. Four genes with conceivable relevant biological importance and/or preliminary association in other populations (unpublished results) to the phenotype in question were included in the study (*ADIPOQ*: rs2241766, *COL22A1*:rs2292927, *LEPR*: rs1137100 and rs8179183, and *MMP10*:rs470154).

**Table 1:** Candidate gene information

Nr	Gene	dbSNP^1^	Location	Allele change	References
1	*ADIPOQ*	rs2241766	Exon 2	T/G	[51]
2	*CILP*	rs2073711	Exon 8	C/T	[34]
3	*COL9A2*	rs137853213	Exon 19	C/T	[10,47]
4	*COL9A3*	rs61734651	Exon 5	C/T	[11]
5	*COL11A2*	rs1799907	Intron	T/A	[52,53]
6	*COL22A1*	rs2292927	Exon 3	C/T	-*
7	*IL1A*	rs1800587	Promoter	C/T	[12,13]
8	*IL1B*	rs1143634	Exon 5	C/T	[12]
9	*IL1F10*	rs3811058	Exon 2	T/C	[54]
10	*IL6*	rs1800797	Promoter	G/A	[23]
11	*IL6*	rs1800796	Promoter	G/C	[23]
12	*IL6*	rs1800795	Promoter	G/C	[23]
13	*LEPR*	rs1137100	Exon 4	A/G	[55]
14	*LEPR*	rs8179183	Exon 12	G/C	[55]
15	*MMP9*	rs17576	Exon 6	G/A	[56]
16	*MMP10*	rs470154	Intron	A/C	-*
17	*SKT*	rs16924573	Intron	G/A	[31]
18	*THBS2*	rs9406328	Intron	T/C	[56]
19	*VDR*	rs10735810	Exon 9	T/C	[8,57]

^1 ^Names as they appear in NCBI SNP data *Unpublished results.

### SNP genotyping

We isolated DNA from frozen whole blood and performed the genotyping using a single base extension fluorescent method, the SNaPshot Multiplex Kit (Applied Biosystems). PCR amplification and SNaPshot extension reactions were both designed to have the same annealing temperature. The SAP/ExoI purified PCR product was processed according to the manufacturer's protocol with the exception of the halved amount (2.5 μl) of SNaPshot Ready Reaction Mix. We carried out analysis using the ABI3031xl Genetic Analyzer (Applied Biosystems), and determined the genotypes using GeneMapper software (Applied Biosystems). We validated the genotype results from a randomly chosen 10% of samples for each SNP by direct sequencing, using the abovementioned genetic analyzer with BigDye chemistry from the same manufacturer. Genotyping was blinded towards MRI status.

### Statistical Analysis

We tested the potential deviation from the Hardy-Weinberg equilibrium (HWE) using the chi-square test. Prior to polymorphism association, the possible effect of gender, BMI and height on the DD status was analyzed using the SAS program (V 9.2). Crude and adjusted ORs and their 95% confidence intervals (CIs) were calculated using logistic regression with SAS. The dependent variable was DD phenotype and independent variables were genotype, allele or haplotype. Haplotypes were first estimated by SNPStats [19] and then statistically reconstructed from population genotype data, using the PHASE program and the Markov-chain method for haplotype assignments [20]. In candidate gene association studies, the questions are more specific than in whole genome association studies. Therefore, in the setting of existing a priori hypotheses, multiple testing corrections were only accounted for in genes with multiple SNPs. The Bonferroni-Holm method was used for multiple testing corrections [21].

## Results

### Clinical results

Of the 538 subjects in this study, 45.7% (N = 246) did not have DD, whereas 54.3% (N = 292) had DD. Of the 292 individuals with DD, 51.4% (N = 150) had moderate DD as defined in the method section. The remaining 142 individuals had degenerative findings but were not included in the analysis. Height and BMI were associated with DD (Table 2), but after adjustment for height, BMI was no longer associated with DD. DD was more frequent among men (32.1%) than women (24.9%), but the difference was not statistically significant when adjusted for height.

**Table 2:** Characteristics of study population at the age of 19

	**Subjects without DD**	**Subjects with DD**
	
N	246	150
Gender (females, %)	66.3	52.0
Height (mean; cm)^1^	167.8	172.3
BMI (mean; kg/m^2^)^2^	22.6	23.4

**^1) ^**p = < 0.0001, **^2)^**p = 0.0384

### Genetic associations

Overall, genotyping of all the subjects from the selected 19 SNPs was 98% successful. All the genotype frequencies analyzed were in HWE. Associations were observed in three of the 16 genes tested. Table 3 presents the genotype frequencies and respective ORs for individual SNP. The strongest associations with DD were observed for polymorphisms in *IL6 *(Table 3). The *IL6 *rs1800795 G-allele was associated with the degenerative phenotype in an additive manner; GC-genotype, OR 1.51 [0.92-2.47] and GG-genotype, OR 2.09 [1.14-3.85]. Minor allele (G) frequencies were 0.490 and 0.398 in subjects with and without DD, respectively (OR 1.45 [1.07-1.96]). The rs1800797 was similarly associated with the DD phenotype, suggesting an additive model (GA: OR 1.55 [0.95-2.54]), GG: OR 1.85 [1.01-3.39]), compared to the AA-genotype. G-allele frequencies were 0.490 and 0.409 among subjects with and without DD, respectively (OR 1.37 [1.02-1.85]). Subsequent haplotype analyses resulted in an association between the GGG haplotype (from rs1800797, rs1800796 and rs1800795, respectively) and DD (OR 1.48 [1.09-2.01]) (Table 4). From this haplotype, only one SNP (rs1800796) did not individually associate with DD. The effect of the GGG haplotype was additive, while the most frequent haplotype irrespective of the MRI findings was AGC.

**Table 3:** Genotype frequencies of the analyzed SNPs

			Subjects without DD*			Subjects with DD*			
					
Gene	SNP	Minor allele	11	12	22	11	12	22	OR (95% CI)
*ADIPOQ*	rs2241766	G	94.3	5.7		88.6	11.4		NS
*CILP*	rs2073711	T	31.7	49.8	18.5	24.0	54.0	22.0	NS
*COL9A2*	rs137853213	T	96.7	3.3		96.0	4.0		NS
*COL9A3*	rs61734651	T	78.8	20.8	0.4	85.3	14.7	0.0	NS
*COL11A2*	rs1799907	A	53.2	40.4	6.4	50.7	42.9	6.4	NS
*COL22A1*	rs2292927	T	59.1	35.2	5.7	59.3	37.0	3.7	NS
*IL1A*	rs1800587	T	43.1	46.3	10.6	42.7	44.7	12.7	NS
*IL1B*	rs1143634	T	57.0	36.9	6.2	54.7	35.3	10.0	NS
*IL1F10*	rs3811058	C	81.2	17.6	1.2	83.3	16.0	0.7	NS
*IL6*	rs1800797	G	35.4	47.3	17.3	24.8	52.4	22.8	1.37 [1.02 - 1.85]^1^
*IL6*	rs1800796	C	93.8	6.2		94.0	6.0		NS
*IL6*	rs1800795	G	36.5	47.5	16.0	25.5	51.0	23.5	1.45 [1.07 - 1.96]^2^
*LEPR*	rs1137100	G	39.6	49.8	10.6	48.0	38.7	13.3	NS
*LEPR*	rs8179183	C	78.0	19.2	2.9	80.5	18.8	0.7	NS
*MMP9*	rs17576	G	37.1	45.7	17.1	37.6	46.3	16.1	NS
*MMP10*	rs470154	T	90.6	9.4		90.0	7.9	2.1	NS
*SKT*	rs16924573	A	93.4	6.6		98.0	2.0		0.26 [0.07 - 0.96]^3^
*THBS2*	rs9406328	T	52.5	41.8	5.7	59.5	31.8	8.8	NS
*VDR*	rs10735810	T	45.1	48.5	6.4	54.3	37.9	7.9	NS

^1) ^log-additive model, corrected p = 0.074 ^2) ^log-additive model, corrected p = 0.042 ^3) ^p = 0.023

*Values are expressed as percentages

**Table 4:** Frequencies of *IL6 *haplotypes and diplotypes

	Subjects without DD		Subjects with DD			
			
	n	%	n	%	OR [95% CI]	*P*-value
AGC	287	59.1	151	50.7	1.00	
GGG	177	36.4	136	45.6	1.48 [1.09 - 2.01]	0.0122
GCG	15	3.1	9	3.0	0.88 [0.37 - 2.12]	
other	7	1.4	2	0.6	-	
						
AGC/AGC	86	35.0	37	24.7	1.00	
GGG/AGC	102	41.5	71	47.3	1.61 [0.97 - 2.67]	0.0650
GGG/GGG	33	13.4	30	20.0	2.22 [1.17 - 4.22]	0.0152
GCG/AGC	10	4.1	5	3.3	1.01 [0.31 - 3.23]	
GGG/GCG	5	2.0	4	2.7	1.17 [0.28 - 4.80]	
GGC/AGC	3	1.2	1	0.7	0.70 [0.07 - 7.28]	
other	7	2.9	2	1.3	-	

The GA genotype of the *SKT *(*KIAA1217*) polymorphism rs16924573 was associated with a reduced risk of DD (OR 0.27 [0.07-0.96], p = 0.024). The frequency of the GA genotype was lower among subjects with DD (2.0%), compared to subjects without DD (6.5%). The *CILP *SNP rs2073711 was associated with DD, but only among women. The CT/TT genotypes, compared to the CC genotype, resulted in an OR of 2.04 [1.07-3.89], and p = 0.025 (corrected p = 0.05). We did not observe statistically significant associations between the other SNPs and DD.

## Discussion

Disc degeneration is described as a common multifactorial and multigenic condition [3]. At least 14 genes have been shown to associate with DD, although their specific effects are not known. In this study, *IL6, SKT *and *CILP *were associated with moderate DD in a sample population of young adults who had their MRI taken at a mean age of 21 years.

*IL6 *is a pleiotropic cytokine which is expressed in many cells and is one of the most important mediators of inflammatory reactions in humans [22]. The role of *IL6 *and in particular the effect of three common promoter polymorphisms (rs1800797, rs1800796, rs1800795) has been studied previously for the presence of sciatica [23], but also in relation to DD among 12- to 14-year-old children [15]. In our population of young adults rs1800797 and rs1800795 were associated with moderate DD. The G allele of both rs1800797 and rs1800795 was the risk allele for DD (OR 1.37 [1.02-1.85] and 1.45 [1.07-1.96], respectively).

The role of *IL6 *was further strengthened with haplotype analyses of the promoter SNPs. *IL6 *haplotype GGG was associated with moderate DD (OR 1.48 [1.09-2.01]). In addition, the risk for moderate DD was higher for the carriers of the GGG/GGG diplotype (OR 2.22 [1.17-4.22]). This finding is supported by an observation that, among sciatica patients, GGGA was the risk haplotype (OR 4.80 [1.59-14.45]) [23]. Furthermore, the GGGA haplotype was a predisposing factor for future sciatica symptoms [24]. In a study of young Danish children (aged between 12 and 14) a GCG haplotype was associated with early DD (OR 6.46 [1.61-26]) [15]. This association was present only in females, and the population with DD was small, consisting of 68 individuals. The two most common haplotypes observed in that study were in accordance with the ones we observed in ours (AGC and GGG), indicating that there are no major differences in genotype frequencies between the two study populations.

Functional analyses have shown that the production of *IL6 *is higher in individuals carrying the GG genotypes for rs1800797 and rs1800795 variations [25]. It has also been reported that the three common promoter polymorphisms influence the transcription of *IL6 *with the G allele by each being responsible for the increased overall transcription of *IL6 *[26]. Overproduction of IL6 has been associated with a spectrum of age-related conditions, including atherosclerosis, peripheral vascular disease, coronary artery disease, and osteoporosis [27]. It is interesting to note that the same common *IL6 *promoter variants are also associated with the most severe forms of distal interphalangeal osteoarthritis [28]. Osteoarthritis and DD share pathological and clinical features, even though the extent to which the etiological determinants of these diseases' processes are similar is not known [29].

The association of the *SKT *rs16924573 variant with DD has not been previously observed. In our population, carriers of the minor allele A, which is a rare allele in the Finnish population, were at a reduced risk of DD. The importance of *SKT *in development of caudal intervertebral discs (IVD) in mice has been established previously [30]. The mice with homozygotely trapped *SKT *gene produce a deformed "*kinky tail" *phenotype - seemingly due to nucleus pulposus changes - during adulthood [30]. These observations led to the first study with human lumbar disc herniation patients characterized by sciatica [31]. In the study an association of *SKT *rs16924573 G allele with lumbar disc herniations was found (OR 1.34 [1.14-1.58]) [31]. DD, phenotype of the present study, is believed to be an important intermediate stage in symptomatic disc disease [32], which is often characterized by disc herniations either with or without sciatic pain radiating from the back to the leg [33]. Our current finding is in line with the earlier findings, however, the differences between the phenotypes in these studies need to be noted. These findings provide some support to the hypothesis that *SKT *could have long-term importance in the onset of symptomatic disc disease by making the discs more vulnerable for DD.

In addition, an association between the *CILP *variant and DD was observed. *CILP *encodes the cartilage intermediate protein, which inhibits the transforming growth factor β1- (TGF-β1) mediated induction of extracellular matrix proteins through direct interaction with TGF-β1 [34]. In the present study, the T allele was associated with an increased risk of DD. The TT/CT genotype compared to the CC genotype showed an OR of 2.04 [1.07-3.89]. The association between *CILP *and DD was, however, only found in females. This finding is in contrast with previously reported associations among Japanese subjects, which indicated the C allele as the risk allele for symptomatic lumbar disc disease [34] as well as for lumbar DD [35,36]. However, a replication study in Finnish and Chinese populations did not find an association between *CILP *and symptomatic disc disease or MRI-defined DD [37]. Interestingly, the C allele was associated with reduced risk of knee OA [38], which is in accordance with our findings that the T allele, and not the C allele, is the risk allele. Although it was shown in functional studies that the C allele is responsible for increased binding and inhibition of TGF-β1 [34], the discrepancies in the association studies could simply be due to the difference in allele frequencies between European and Japanese populations http://www.ncbi.nlm.nih.gov/snp.

In our population, both men and women with DD were significantly taller than those without DD. The association between body height and herniated lumbar disc has previously been contradictory [39,40]. In our earlier study, height was associated with DD on MRI among 12- to 14-year-old Danish children (N = 220) [15]. In another study among middle-aged women living in the UK (N = 796), individuals with radiographically defined DD progression over a 9-year period were taller than individuals without DD progression, but the difference (0.7 cm) was not statistically significant [41]. However, in a recent large cohort study (N = 13, 282) body height recorded at baseline was associated with higher likelihood of back surgery and low back pain during the 12-year follow-up. Although the associations reached statistical significance only among males, a similar trend was observed among females as well [42].

One possible mechanism for the role of body height as a risk factor is lumbar disc size. Larger discs might be predisposed to DD due to greater diffusion distances of nutrients into the centre of the nucleus pulposus [43]. Furthermore, higher discs might be at an increased risk of mechanical failure under external loading [44]. Finally, facet joint alterations have been found to be more frequent in taller individuals with lumbar disc herniation [45].

To date there have been many reported genetic associations in relation to DD, yet only a few have been replicated in different populations. We were not able to detect any significant associations for the majority of the polymorphisms that have been previously implicated in DD. This discrepancy may be due to differences in the definitions of the DD phenotype, sample characteristics, or true differences in the prevalence of polymorphisms among ethnic populations. The majority of these studies have been mainly conducted in adult populations, and only a few have been performed specifically in younger populations. It is well possible that a particular gene is associated with DD at only a particular age; for instance, a 5A/6A promoter polymorphism of the *MMP3 *gene associated with more degenerative findings in the elderly with a mean age of 73 than in younger subjects with a mean age of 21.4 [46]. It has also been demonstrated that the Trp2 allele of *COL9A2 *is a significant age-dependent risk factor for the development and severity of DD, increasing the risk of DD in the 40 to 49 year age group in the Trp2 carriers [47].

The strength of our study is the young age of the participants of this population-based birth cohort. The effects of environmental factors are most likely not so evident, whereas genetic factors may play a more important role in comparison to adult populations. Furthermore, in order to avoid any bias, our genotypes were defined a priori without any knowledge of the results of MRI readings. Our DD phenotype was also defined a priori before the genetic analyses and three specialists evaluated the MR images independently. Despite the initial large number of MRI scans, our sample study size is still fairly modest for studying multifactorial and multigenic conditions. A further limitation in the study was that only one SNP was included for the majority of the genes. For a comprehensive evaluation of the gene, a more extensive SNP analyses across the gene regions would be needed.

DD is a common disorder and is believed to be an important intermediate stage in symptomatic disc disease [32]. Several recent studies suggest that DD is associated with low back symptoms [48-50]. The findings indicate that a higher degree of lumbar DD is related to a higher likelihood of symptoms, and the presence of moderate DD or DD at multiple levels increases the likelihood of pain [50]. Therefore, the clinical relevance of genetic studies exploring the pathomechanisms of DD is indisputable.

## Conclusions

In summary, five variations of the 19 selected candidate SNPs were associated with susceptibility to DD in our study. Our results support the role of *IL6*, *SKT *and *CILP *in DD. Furthermore, we were able to show the associations in a population of young adults. Overall, genetic association studies provide information about specific gene variants on disease occurrence, but interpreting their results has proven to be a challenge [29]. The lack of association of well-known variations observed here does not necessarily contradict previous reporting. Instead our results emphasize the complexity of DD, and the important role of further studies in populations with different ethnic and age backgrounds.

## Competing interests

The authors declare that they have no competing interests.

## Authors' contributions

AK, PE and UK participated in analysis and interpretation, design and writing. JT, ID, JN, RBS, OT, SS and AO participated in analysis and interpretation and critical revising. PYPK, YQS, KMCC, DC, L-AK and M-RJ participated in conception, design and some in critical revising. JK and MM participated in conception, design of the study, writing, critical revisions and supervision. All authors have approved the final version of the manuscript to be submitted.

## Pre-publication history

The pre-publication history for this paper can be accessed here:

http://www.biomedcentral.com/1471-2350/12/153/prepub
